# The Impact of a Web-Based Course Concerning Patient Education for Mental Health Care Professionals: Quasi-Experimental Study

**DOI:** 10.2196/11198

**Published:** 2019-03-01

**Authors:** Anna Laine, Maritta Välimäki, Eliisa Löyttyniemi, Virve Pekurinen, Mauri Marttunen, Minna Anttila

**Affiliations:** 1 Department of Nursing Science University of Turku Turku Finland; 2 School of Nursing Hong Kong Polytechnic University Hong Kong China (Hong Kong); 3 Department of Biostatistics University of Turku Turku Finland; 4 University of Helsinki Helsinki Finland; 5 Helsinki University Hospital Helsinki Finland

**Keywords:** internet, online education, mental health, continuing education

## Abstract

**Background:**

Continuing education has an important role in supporting the competence of health care professionals. Although Web-based education is a growing business in various health sectors, few studies have been conducted in psychiatric settings to show its suitability in demanding work environments.

**Objective:**

We aimed to describe the impact of a Web-based educational course to increase self-efficacy, self-esteem, and team climate of health care professionals. Possible advantages and disadvantages of the Web-based course are also described.

**Methods:**

The study used nonrandomized, pre-post intervention design in 1 psychiatric hospital (3 wards). Health care professionals (n=33) were recruited. Self-efficacy, self-esteem, and team climate were measured at 3 assessment points (baseline, 8 weeks, and 6 months). Possible advantages and disadvantages were gathered with open-ended questions at the end of the course.

**Results:**

Our results of this nonrandomized, pre-post intervention study showed that health care professionals (n=33) had higher self-efficacy after the course, and the difference was statistically significant (mean 30.16, SD 3.31 vs mean 31.77, SD 3.35; *P*=.02). On the other hand, no differences were found in the self-esteem or team climate of the health care professionals before and after the course. Health care professionals found the Web-based course useful in supporting their work and relationships with patients. The tight schedule of the Web-based course and challenges in recruiting patients to use the patient education program with health care professionals were found to be the disadvantages.

**Conclusions:**

Web-based education might be a useful tool to improve the self-efficacy of health care professionals even in demanding work environments such as psychiatric hospitals. However, more studies with robust and sufficiently powered data are still needed.

## Introduction

### Background

Mental health settings are demanding work environments for health care professionals [1,2]. Mental health care professionals in particular face challenging and ethically burdening situations in psychiatric hospitals [3,4]. To deal with these demanding situations, health care professionals need to work effectively [5] and be confident in their professional role [6]. Maintaining competence is an ethical responsibility of health care professionals [7,8], and the capacity to use information technology has been included as a core capacity in health care [9].

Continuing education is an important part of employability and personal development [10], and the costs of continuing education are remarkable in Europe [11]. The previous studies have found that education has an important role in health care professionals’ feelings of competence [12,13]. Other studies have also reported association between education and work-related self-efficacy [12,14,15], general self-efficacy [16], self-esteem [17,18], knowledge level, skills [19], and personal development [20]. Furthermore, high competence [12,21] and personal development [16,22] have been found to have a positive impact on health care professionals’ self-efficacy. For example, Proudfoot et al [23] found in their study that having less nonclinical staff was associated with better team climate. On the contrary, low self-esteem seems to be an explanatory variable when it comes to burnout among health care professionals [24].

Web-based learning has become a rapidly growing educational method [25]. Besides catering to different learning styles [26], it offers flexible [27] and effective education for health care professionals [28-31]. Web-based education requires the capacity to use a computer [32] and a positive attitude toward computers [33]. The impact of Web-based learning methods is still less often assessed in mental health care settings than it is in other health care settings [29]. Web-based learning has been used in practice, for example, to implement suicide prevention guidelines [34], to decrease the number of patient aggressive events and physical restrictions used in psychiatric hospitals [35], and to support health care professionals’ engagement in personality difficulties [36]. On the other hand, the use of computers in supporting the knowledge level of health care professionals, changes in attitudes, and work habits has been doubted, not only regarding education but in mental health services altogether [37-39]. It has been questioned, for example, whether real learning requires face-to-face contact [30]. At the same time, a wide range of Web-based interventions have been developed for persons with special mental health needs [40-43]. A lack of computer skills [44,45], a lack of confidence in computer use [46], or the wide range of diverse Web-based mental health interventions [47] might increase concerns of the benefit of Web-based e-learning methods in continuing education.

### Objectives

Earlier studies have shown that higher education [16,48], theory-based training [49], and communication skills training [50] have positive impacts on the self-efficacy of health care professionals. In addition, Web-based education has been found to have a positive effect on health care professionals’ knowledge, attitudes, and practices [51]. However, there is still the need to evaluate the impacts of Web-based education [29]. Therefore, the purpose of this study was to describe the impact of a Web-based educational course on the self-efficacy, self-esteem, and team climate of health care professionals. Possible advantages and disadvantages of the Web-based course are also described. In this quasi-experimental study, we hypothesize that the Web-based course can be a beneficial and usable learning method to support health care professionals’ self-efficacy and self-esteem and support a positive team climate among health care professionals. In addition, the health care professionals’ perception of advantages and disadvantages of the course are described.

## Methods

### Design

A nonrandomized, pre-post intervention design was used. This design is suitable for our purpose as it is used to evaluate the benefits of specific interventions as preintervention and postintervention measurements with nonrandomly selected control groups [52]. A qualitative approach was also used to describe the health care professionals’ feedback on the Web-based course [53].

### Setting

The study was conducted on 3 closed psychiatric inpatient wards (a total of 41 beds) at 1 psychiatric hospital in southern Finland. The wards treated adult patients with serious mental disorders such as schizophrenia, which is the most common diagnosis for patients treated in Finnish psychiatric hospitals [54]. Moreover, 2 wards were rehabilitation wards and 1 was an acute ward.

### Participants

Participants were health care professionals from the 3 wards working in multi-professional teams (altogether 46 health care professionals: 40 nurses, 3 psychologists, 1 occupational therapist, and 2 not known), which is essential in ensuring good patient care [55]. Nurses make up the largest professional group in current psychiatric health services in Finland. In 2014, there were altogether 258,567 people working in municipal health and social care including 46,446 nurses and 89,800 practical nurses or people working in similar occupations [56].

Inclusion criteria for health care professionals were that they worked in the selected wards, participated in the Web-based course targeted for the health care professionals, were Finnish-speaking, and were willing and able to participate in the study. Temporary workers in the ward were excluded.

### Recruitment and the Follow-Up

An information meeting was held at the hospital concerning the Web-based course and the study, and the course was offered to all health care professionals (N=46) from the 3 study wards. All health care professionals were recruited to participate in the study after the meeting.

**Table 1:** The schedule of recruitment, intervention, and assessments.

Schedule of the study	Study period					
	Recruitment (March 2015)	Baseline (April 2015)	Post intervention			Closeout (6 months)
			Month 1	Month 2	8 weeks	
Eligibility screen	X^a^	N/A^b^	N/A	N/A	N/A	N/A
Informed consent	X	N/A	N/A	N/A	N/A	N/A
Web-based course	N/A	N/A	X	X	N/A	N/A
Background information	N/A	X	N/A	N/A	N/A	N/A
Self-esteem measurement	N/A	X	N/A	N/A	X	X
Self-efficacy measurement	N/A	X	N/A	N/A	X	X
Team climate measurement	N/A	X	N/A	N/A	X	X
Feedback of Web-based course	N/A	N/A	N/A	X	N/A	N/A

^a^X: applicable.

^b^N/A: not applicable.

Baseline data were collected in April 2015, and follow-ups were conducted after 8 weeks and 6 months using an online questionnaire. A link to the online questionnaire was sent to the health care professionals before the 3 measurement points. One researcher (VP) monitored data collection and sent reminder messages to the health care professionals. The timeline of participation is presented in Table 1 (modified based on the study by Chan et al [57]).

### Intervention

Previous studies have shown that health care professionals have difficulties in supporting patients in computer use, especially in using Web-based patient education [39,58]. We assumed that if nurses were more familiar with online websites and Web-based education, it might encourage nurses to support patients’ use of computers [59]. Therefore, a Web-based course about patient education was organized for health care professionals to enhance their capacity to support patients in Web-based education.

The Web-based course comprised 4 modules: (1) patient education—methods, effectiveness, and ways to put it into practice, (2) initializing of information technology in mental health care, (3) patient education in practice, and (4) practical training in patient education with a Web-based program. As an example of patient education, we used the website MentalNet, whose quality has been approved by the Health On the Net Code of Conduct to certify that it is a trustworthy and reliable medical website [60]. The MentalNet website has been developed for professionals, patients with psychosis (International Statistical Classification of Diseases and Related Health Problems, tenth revision, codes F20-F29 [61]), and their family members. It includes information about mental illness, treatment, well-being, patients’ rights, daily life, links to other websites, a discussion forum, and a question and answer column [62]. More detailed information about the course is provided in Table 2 (modified based on Template for Intervention Description and Replication [63]).

### Outcomes

In this study, self-reported instruments were chosen to be used because we were interested in getting the health care professionals’ own perspective [67]. Self-reported instruments are useful when measuring respondents’ personal opinions and beliefs and delivering questionnaires electronically without interpersonal contact [68].

#### Primary Outcome

Self-efficacy was measured with the General Self-Efficacy Scale (GSE). This instrument is designed to measure general self-efficacy as a person’s capacity to trust his or her own ability to survive in new or difficult situations in life rather than situation-specific self-efficacy [69]. The instrument includes 10 questions (4-point Likert scale). The sum score of the answers ranges from 10 to 40; a lower score represents poorer coping on a daily basis. The instrument is widely used in studies concerning health care professionals [16,70,71]. Psychometric properties of the GSE have been examined in 25 countries; the Cronbach alpha has varied from .75 to .91 [72]. In this study, the Cronbach alpha was .82.

#### Secondary Outcomes

##### Self-Esteem

Self-esteem was measured using the Rosenberg Self-Esteem Scale (SES) [73]. The instrument is designed to measure global self-esteem. SES is widely used in educational [18] and other types of studies concerning health care professionals [24,74-76]. It includes 10 items with a 4-point Likert scale. The sum score of the answers ranges from 10 to 40. Having a higher score indicates a higher level of self-esteem. In a review by Schmitt and Allik [77], the data of the SES from 53 countries were compared. Internal consistency was found to be good (Cronbach alpha .80, range .45 to .90). In these data, the Cronbach alpha was .86.

**Table 2:** Description of the intervention.

Categories	Description of the intervention
Name: patient education in mental health work	Educational intervention for health care professionals in psychiatric hospitals to deliver patient education
Rationale and theory [64-66]	Health care professionals’ education provides competence and skills for professionals to deliver patient education. Patient education is an important component of care of people with severe mental health disorders. It enables patients to cope more effectively with the illness, which has a positive effect on their well-being. Educational intervention focuses on professional knowledge transfer and health care professionals’ behavioral changes. It provides reasoning for patient education and Web-based services, knowledge of the effectiveness of patient education, and methods to deliver it
Materials	Information about evidence-based research, a written information package, and MentalNet website usable with user account and password (also accessible via an URL link)
Procedures	Individual and group studying and exercises based on health care professionals’ own time schedules; individual and interactive exercises, including a reflective diary and discussions on the forums; and delivery of 5 practical patient education exercise sessions with a patient using the MentalNet website in which patients had their own user account and password
Providers	Trained teachers, tutors, and researchers with a background in mental health provided feedback to the health care professionals after the completed exercises and answered any emerging questions. A course coordinator answered emerging questions and informed about the phases of the course
How	PowerPoint presentations, reading material, and a manual on how to proceed with patient education in clinical practice
Where	On the Moodle learning portal hosted by the University of Turku, usable with personal user accounts and passwords
When and how much	The course consisted of 5 phases (3 European Credit Transfer and Accumulation System credits, 72 hours), and the length of the course was 2 months
Tailoring and modifications	The course was held over a 9-month period due to vacations and health care professionals working in 3 shifts in the hospital

##### Team Climate

Team climate was measured with the Team Climate Inventory (TCI) [78] Finnish version [79]. The TCI is based on West’s theory of innovation [80], and it includes 4 subscales with a varying number of items. The 4 subscales are (1) participative safety (12 items), (2) support for innovation (8 items), (3) vision (10 items), and (4) task orientation (8 items). The first 2 subscales are evaluated with a 5-point Likert scale and the next 2 subscales with a 7-point scale. The TCI has been found to be a reliable measurement. In a previous study involving health care professionals in Finnish mental health care, the Cronbach alpha of the Finnish version of the TCI was as follows: (1) participative safety: .88, (2) support for innovation: .87, (3) vision: .95, and (4) task orientation: .91 [81]. In this study’s data, the Cronbach alpha values were as follows: (1) participative safety: .91, (2) support for innovation: .89, (3) vision: .95, and (4) task orientation: .92.

##### Advantages and Disadvantages of the Web-Based Course for Health Care Professionals

Health care professionals’ perceptions about advantages and disadvantages of the Web-based course were collected via a questionnaire in the course platform by using the following open questions: (1) how does the Web-based course support the professional skills of mental health care professionals? and (2) what were the advantages and disadvantages of the course?

#### Background Information

Information about the health care professionals’ gender, age, level of work experience, and information about their internet use, purposes, skills, and attitudes were collected. Health care professionals’ internet use was described using an adapted measure by Choi and DiNitto [82]: “Have you ever used the internet?" (1) "No, I have never used it" (never user), (2) "I have used it before but not currently" (previous user), and (3) "Yes, I am a current user" (current user). Furthermore, the purpose of the health care professionals’ internet use was explored. Health care professionals were asked to select what kind of activities they conducted on the internet. Options included research health-related information; research information about other topics or issues of interest; send/receive email; buy products online; conduct banking online and/or pay bills; read news, papers, magazines, and books online; play games online; watch videos (including YouTube); use social networking or dating sites (eg, Facebook and Match.com); and other (they were asked to specify) [82]. Activities in which a health care worker would “communicate with health professionals about health-related issues” or “communicate with other users about health-related issues” were added to the original list [83]. Internet/computer skills were evaluated in an item with a 5-point Likert scale: “Your computer/internet-skills are” (1=very good to 5=very poor), whereas attitudes against the computer/internet were examined using an item with another 5-point Likert scale (1=very positive to 5=very negative).

### Sampling and Recruitment of the Participants

Consecutive sampling was used to recruit all the health care professionals who worked in these 3 wards and who participated in the Web-based e-course targeted for the health care professionals. This sampling method was suitable for our quasi-experimental study, as we aimed to recruit all possible study participants accessible at the time of data collection [53].

### Data Analysis

Numerical variables are summarized with a median, mean, and SD, whereas categorical variables are reported with counts and percentages. The total sums for scores were calculated. Self-efficacy, self-esteem, and factors of team climate were all analyzed with hierarchical linear mixed models for repeated measures for all 3 time points (baseline, 8 weeks, and 6 months). The model was adjusted for age, gender, and work experience. A compound symmetry covariance structure was used for repeated measures. All statistical tests were performed as two-sided with the statistical significance level set at .05. The analyses were performed using the SAS System, version 9.4 for Windows (SAS Institute Inc, Cary, NC, US).

Cohen *d* was assessed at baseline and 6 months to determine the effect size of the intervention on measured outcomes. An effect size of 0.2 is considered small, 0.5 is medium, and 0.8 is a great effect size [84]. An online calculator was used for the analyses [85].

A qualitative analysis was conducted by 2 authors (AL and MA). Writings of health care professionals were collected from the Moodle learning portal. Similarities in the answers were searched for based on the study questions and were classified into categories [86]. The texts answering the question asking how the Web-based course supports professional skills were combined with the answers about advantages of the course because the answers were very similar.

### Ethics

Ethical permission for the research was obtained from the ethics committee of the Hospital District of Southwest Finland (ETMK:40/1801/2015). Permission for data collection was obtained from the research permission committee of the city (2015-002638).

After providing oral and written information about the study, an information letter and informed consent forms were sent via email to the health care professionals of the wards. The health care professionals were asked to print 2 copies and sign them, after having approximately 2 weeks to decide if they wanted to participate in the study or not. Of them, 1 researcher (AL) collected 1 signed consent form from each health care professional when visiting the wards and left the duplicate with the health care professional. Participation was voluntary, and withdrawal was allowed at any time without needing to give a reason. The data were handled by a research group. It used anonymous ID codes [87].

## Results

### Participant Flow

**Figure 1:**
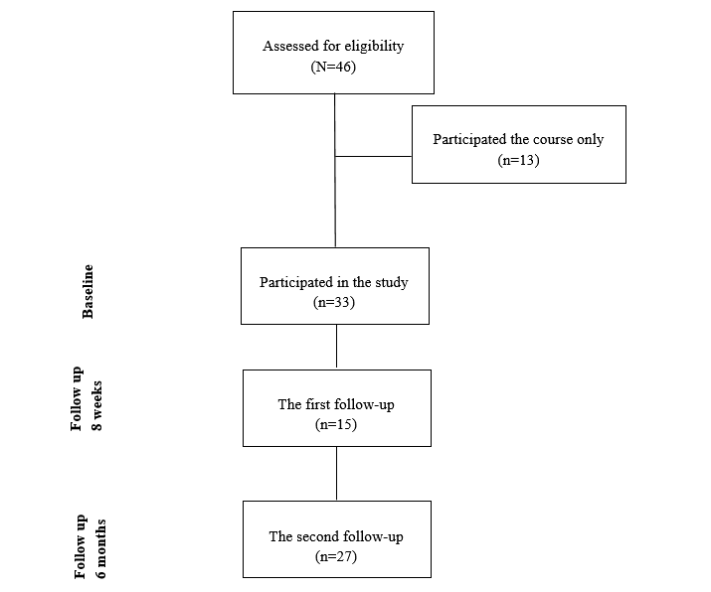
Flow diagram of the participants.

Out of 46 possible Web-based course participants, 13 (28%) health care professionals decided to participate only in the Web-based course and 33 (72%) participated both in the study and online course. Out of them, 15 (45%) participated in the first follow-up and 27 (82%) in the second follow-up. A description of the flow diagram of the participants is presented in Figure 1.

### Description of Participants

The study sample consisted of 33 health care professionals, 24 of them were females and 9 were males. Their mean age was 41 years (range 25-61), and their mean working experience in mental health care was 14 years (range 2-40). All health care professionals used the internet (33/33, 100%). Over half (21/33, 64%) estimated that they have very good or good computer skills and two-thirds (27/33, 82%) had very positive or positive attitudes toward the internet. Health care professionals chose all options for internet use that were related to them. They used the internet for banking, emailing, searching for health care knowledge, and reading news or books (Table 3). In addition, 4 health care professionals also used the internet for music streaming, searching for information on public transportation, discussion purposes, or for work.

### The Impact of the Web-Based Course on Health Care Professionals’ Self-Efficacy, Self-Esteem, and Team Climate

The impact of the Web-based course on health care professionals’ self-efficacy, self-esteem, and team climate was measured at 3 time points. The analysis showed that health care professionals’ self-efficacy scores increased between baseline (mean 30.16, SD 3.31) and 8 weeks (mean 31.53, SD 2.82), and even further at 6 months (mean 31.77, SD 3.35). The difference between baseline and 6 months measurements was statistically significant (*P*=.02) with an effect size of 0.48 (Cohen *d*).

On the contrary, no statistical differences were found in health care professionals’ self-esteem during the 6-month study period. In addition, no significant changes were found in health care professionals’ team climates or its subscales (participative safety: *P*=.82, support for innovation: *P*=.81, vision: *P*=.48, and task orientation: *P*=.85; see Table 4).

**Table 3:** Internet skills, attitudes toward the internet, and internet use of health care professionals.

Characteristic (n=33)		n (%)
**Computer/internet skills**		
	Very good	7 (21)
	Good	14 (42)
	Neither good nor bad	9 (27)
	Fairly poor	2 (6)
	Poor	1 (3)
**Attitudes toward computers/internet**		
	Very positive	13 (39)
	Positive	14 (42)
	Neither positive nor negative	5 (15)
	Negative	1 (3)
	Very negative	0 (0)
**Purpose of internet use**		
	For banking online and/or to pay bills	33 (100)
	Send/receive email	32 (97)
	Research health-related information	30 (91)
	Read news, papers, magazines, and books online	30 (91)
	Research information about other topics of interest	28 (85)
	Buy products online	24 (73)
	Watch videos	22 (67)
	Use social networking or dating sites	19 (58)
	Play games online	7 (21)
	Communicate with health professionals about health-related issues	5 (15)
	Other	4 (12)
	Communicate with other users about health-related issues	3 (7)

**Table 4:** Hierarchical linear mixed models of self-efficacy, self-esteem, and factors of team climate at baseline, 8 weeks, and 6 months.

Measurement		Time point						*F* (*df*)	*P* value	Cohen *d*
		Baseline		8 weeks		6 months				
		n	Mean (SD)	n	Mean (SD)	n	Mean (SD)			
Self-efficacy		32	30.16 (3.31)	15	31.53 (2.83)	26	31.77 (3.35)	4.59 (2)	.02^a^	0.48
Self-esteem		33	34.00 (4.62)	15	33.80 (4.93)	26	35.54 (4.01)	1.55 (2)	.23	0.36
**Team climate**							
	Participative safety	33	3.99 (0.57)	15	3.97 (0.46)	27	3.96 (0.46)	0.19 (2)	.82	−0.06
	Support for innovation	32	3.50 (0.59)	15	3.47 (0.66)	27	3.57 (0.57)	0.21 (2)	.81	0.12
	Vision	33	5.41 (0.90)	15	5.10 (0.68)	26	5.38 (0.89)	0.74 (2)	.48	−0.03
	Task orientation	33	5.06 (0.95)	15	4.87 (0.72)	26	4.96 (0.91)	0.16 (2)	.85	−0.01

^a^Statistically significant difference analyzed with hierarchical linear mixed models for repeated measures.

The demographic characteristics of nonparticipating and participating health care professionals in the first follow-up were similar. The mean age of the nonparticipating health care professionals was 41 years (range 30-61), and participating health care professionals had a mean age of 38 years (range 25-60). The mean number of working years was 15 (range 2-38) for nonparticipating health care professionals and 14 years (range 2-40) for participating health care professionals. Out of the 18 nonparticipating health care professionals, 4 (22%) were men, and out of the 15 participating health care professionals, 5 (33%) were men.

### Advantages and Disadvantages of the Web-Based Course

#### Advantages of the Web-Based Course

On the basis of the health care professionals’ descriptions, the advantages of the course were divided into 5 categories: (1) a modern method for patient education, (2) support for health care professionals’ competence, (3) support for discussions between a health care professional and a patient, (4) support for relations between a health care professional and a patient, and (5) support for structural nursing. The advantages and examples of phrases from health care professionals are presented in Table 5.

First, in general, the use of computers was described as a novel way to administer patient care in ward settings. Health care professionals found that the Web-based resources were a modern approach to carrying out patient education, and the website was a new tool they could use for it:

MentalNet opens a new and modern way to realize patient education.

The staff have gained one new way for implementing psychoeducation.

The staff have received a new tool for themselves.

Second, health care professionals described that the Web-based course supported their competence level. They were able to find current information, recall old information, and also learn something new when they used the website together with patients. The course also supported professional skills when health care professionals were able to learn how to use a computer in patient care:

The latest versatile information needed in caring for a patient with psychosis can be found on the website.

While going through MentalNet patient education, I got new information about the latest recommendations myself.

The staff have benefited from the repetition of the psychoeducation content.

Nurses also learn to utilize information technology

Third, the Web-based course supported discussions between health care professionals and patients. Health care professionals considered the themes of the website to be starting points for discussions. The themes were also seen as a checklist when all important topics were discussed through them. Health care professionals described how using the website in discussions helped them to bring up different topics. Patient education meetings were found to be natural moments to also bring up those topics that were otherwise difficult to discuss with the patients:

The program would be a good foundation for psychoeducation. The topics are categorized, so you can address them according to the patient's interests.

Themes are collected as a clear checklist, and it is always possible to go back to something that a patient is wondering about.

There are topics that would have not necessarily been worked through with a patient otherwise (e.g. sexuality, patient’s rights).

Patient education sessions included many natural situations and moments to bring up things in different themes.

Fourth, patient education helped to create, support, and strengthen relationships between patients and health care professionals. Health care professionals described that using the website together with the patient helped them to have good contact with the patients and create relationships with the patients. After using the website, it was also easier to recognize the patients’ needs and understand their symptoms:

The use of MentalNet may make it easier to create a good patient-nurse relationship.

There has been an opportunity to obtain good contact with the patient.

MentalNet provides a foundation for staff to better understand patients’ symptoms, and it makes patients’ situations easier to identify with.

Fifth, the course was found to support structural nursing and the planning aspect of patient education. Health care professionals found that using the website made patient education more systematic. It also strengthened the quality of the patient education when all patients got the same information regardless of which ward or health care professional provided it:

The adoption of patient education has brought more planning.

In addition, it gives staff an opportunity to deal with aspects concerning patients’ health and wellbeing in an even more systematic manner.

All patients get similar information regardless of the ward or nurse, which strengthens the quality of the care.

**Table 5:** Advantages of the Web-based course.

Categories and subcategories		Phrases
**A modern method for patient education**		
	Modern way to realize patient education	“The staff have gained one new way for implementing psychoeducation.”; “MentalNet opens a new and modern way to realize patient education.”; and “MentalNet is a good new method that can be used to provide and maintain patient education to patients of the ward.”
	New tool to realize patient education	“MentalNet has provided a new instrument for implementing patient education to patients treated in the ward.”; “The staff have gained a new tool for implementing psychoeducation.”; and “The staff have received a new tool for themselves.”
**Support for health care professionals’ competence**		
	Current information for health care professionals	“The site provides current information to staff in the same manner as it does to patients.”; “The latest versatile information needed in caring for a patient with psychosis can be found on the website.”; and “Information is collected into themes, which can be easily accessed with a link.”
	Develop and maintain health care professionals’ knowledge	“The theme may even expose nurses to new information also.”; “MentalNet also provides the staff with a lot of well-researched and up-to-date information on mental health issues, which will help to maintain good professional skills.”; and “While going through MentalNet patient education, I got new information about the latest recommendations myself.”
	Repetition of knowledge for health care professionals	“The content resembles things that have already been learned.”; “The staff have benefited from the repetition of the psychoeducation content.”; and “By using MentalNet, it is also possible to recall up to date and trustworthy information, and to possibly gain some new information.”
	Support for health care professionals’ skills in technology	“Nurses also learn to utilize information technology” and “It has given us experience in how computers can be used in patient care.”
**Support for discussions between a health care professional and a patient**		
	Base for discussions with a patient	“MentalNet can be used as a foundation for discussion between nurses and patients.”; “The program would be a good foundation for psychoeducation. The topics are categorized, so you can address them according to the patient's interests.”; and “I believe that MentalNet can be a useful tool for patient education and as a basis for discussions between nurses and patients.”
	Checklist for discussions with the patient	“Themes are collected as a clear checklist, and it is always possible to go back to something that a patient is wondering about.”; “Providing psychoeducational information has become easier; all the details do not have to be memorized by heart and the information is structured in MentalNet.”; and “Themes regarding psychosis are clearly grouped together, and they serve as a good checklist for the professional.”
	Helps to bring various themes into discussions	“There are topics that would have not necessarily been worked through with a patient otherwise (eg, sexuality and patient’s rights).”; “I personally feel that, with the program, it was easier to bring up things with the patient.”; and “It encourages the discussion of health and wellbeing-related topics with the patient: For example, the importance of sleep, nutrition and exercise to wellbeing are not usually covered in patient education as thoroughly as it is in medical treatment and psychosis. Also, it makes it easier to bring up topics and patient system dimensions that are not needed at the moment.”
	Natural moments to discuss different topics	“Patient education meetings were natural moments to bring up different topics with the patient.”; “Patient education sessions included many natural situations and moments to bring up things in different themes.”; and “During the patient education, discussing the most difficult things were easier and more natural for the patient.”
**Support for relations between a health care professional and a patient**		
	Help for a good relationship with a patient	“The use of MentalNet may make it easier to create a good patient-nurse relationship.”; “It has helped to create a good nurse-patient relationship.”; and “For example, creating a new framework for being connected with the patient/patients.”
	Strengthens interaction between a health care professional and a patient	“Using MentalNet created a good moment of interaction with a patient.”; “Strengthens interaction with patients.”; and “There has been an opportunity to obtain good contact with the patient.”
	Increase of understanding of patients’ needs and symptoms	“Recognizing patients’ needs is easier.” and “MentalNet provides a foundation for staff to better understand patients’ symptoms, and it makes patients’ situations easier to identify with.”
**Support for structural nursing**		
	Planning and systematic increase of patient education	“The adoption of patient education has brought more planning.”; “Patients are dealt with more systematically.”; and “In addition, it gives staff an opportunity to deal with aspects concerning patients’ health and wellbeing in an even more systematic manner.”
	Same information for all patients	“All patients get similar information regardless of the ward or nurse, which strengthens the quality of the care.” and “The content stays the same even if the patient changes.”

#### Disadvantages of the Web-Based Course

Disadvantages of the course consisted of 3 categories: (1) factors concerning schedule and working time, (2) factors concerning patients, and (3) factors concerning equipment and environment. Disadvantages and examples of phrases from health care professionals are presented in Table 6.

First, health care professionals described disadvantages concerning schedules and working times. Tight schedules and the hurried implementation of the course were found to be challenging and caused stress. Completing the course and scheduling the patient education meetings were challenging because of shift work and vacation days during the course:

The busy timetable for implementing the course was challenging and caused stress to at least some of the personnel.

Scheduling patient education meetings was challenging because of shift work.

The schedule has been tight, and it was during a period when some of the employees still had winter holidays and some were about to start their summer holidays. This has created some challenges in the implementation.

Second, health care professionals described disadvantages concerning patients. Health care professionals had difficulties finding willing and suitable patients to participate in patient education. Some of the patients also had a lack of computer skills, which complicated the patient education:

There was pressure to hold education meetings and to find willing patients with the cognitive skills to understand the content.

Also, however, using a computer is often difficult for patients. Therefore, we sometimes have needed to start with how to use the computer.

Third, factors concerning the equipment and the environment were considered to be a disadvantage, as health care professionals described the fact that there were only a few computers that could be used for Web-based patient education. Moreover, when health care professionals needed to book a room for himself/herself and a patient, other health care professionals and patients could not use the room during that time:

There are only a few computers available for patient education.

When one nurse stays an hour in a patient education meeting and a group room is occupied for that hour, others have to adapt their work and schedule around the meeting accordingly.

**Table 6:** Disadvantages and examples of phrases from health care professionals.

Categories	Subcategories	Phrases
Factors concerning schedules and working times	Tight schedule of the course	“The busy timetable for the implementation of the course was challenging and caused stress to at least some of the personnel.”; “The schedule of the course was pretty tight, which created challenges in the implementation.”; and “The schedule of this course has added negative pressure to the work.”
	Challenges concerning working times and the course	“Scheduling patient education meetings was challenging because of shift work.”; “It was challenging to fit schedules to one’s own working time.”; and “The schedule has been tight, and it was during a period when some of the employees still had winter holidays and some were about to start their summer holidays. This has created some challenges in the implementation.”
Factors concerning patients	Patients’ unwillingness to participate in patient education	“There was pressure to hold education meetings and to find willing patients with the cognitive skills to understand the content.”; “Some were stressed by patients’ low interest in taking part in the MentalNet patient education.”; and “The drawback for some staff has been patients who are not willing to take part in education/research.”
	Patients’ poor computer skills	“Also, however, using a computer is often difficult for patients. Therefore, we sometimes have needed to start with how to use the computer.”
Factors concerning equipment and environment	Room booked during the patient education	“When one nurse stays an hour in patient education meeting and a group room is occupied for that hour, others have to adapt their work and schedule around the meeting accordingly.”
	Too few computers available	“There are only a few computers available for patient education.”

## Discussion

### Principal Findings

The aim of our small-scale study was to find out how a Web-based course impacts health care professionals’ self-efficacy, self-esteem, and team climate. The results show that health care professionals who participated in the Web-based course focusing on patient education had an increased sense of self-efficacy at baseline and after the course, and again after 6 months. These findings support earlier studies that found that the self-efficacy of health care professionals could be supported by education. Other studies have shown that health care professionals who start their career with a higher educational level seem to have higher self-efficacy than health care professionals with lower education [16,48]. Similar to our intervention, some studies have shown that continuing education for health care professionals with theory-based training [49] and communication training [50] increases health care professionals’ self-efficacy.

In contrast to previous findings, we did not find any significant difference between health care professionals’ self-esteem at different time points. On the basis of a study by Van Eckert et al [18], nurses with an academic education have higher self-esteem than nurses without an academic education. Therefore, it is possible that this kind of short course might not be powerful enough to support self-esteem. Furthermore, no changes were found between health care professionals’ sense of team climate during the course of follow-up. We assumed that team climate would be stronger after the course because being in close cooperation and reflecting on learning experiences together might positively affect team climate [88], and teamwork can increase a sense of belonging [89]. Having a sense of a good team climate is important for nurses’ well-being [90], and this might, in turn, affect patients’ satisfaction levels with care [23]. It is still unclear whether a good team climate can be developed by using technological solutions. Although team climate for health care professionals is an important goal in health services, Web-based learning might not be the prioritized method for this task. However, expectations toward the adoption of technology in health care are positive [9].

In our study, the health care professionals found the Web-based course to be diversely useful. According to their feedback, the Web-based course supported the structure of patient education. Health care professionals were able to utilize the content of the MentalNet website as *a checklist* in conversations with the patients. They also found the website helpful in supporting their professional skills and the structure of nursing activities. Some health care professionals thought that MentalNet supported their relationships with patients because it provided evidence-based knowledge that could be used during challenging discussion topics, such as sexuality. This feedback is useful information for improving relationships between health care professionals and patients on a larger scale—something that has been found to be crucial for treatment adherence, for example [91]. Patient education is an essential part of care for patients with schizophrenia [92]. Therefore, it is important to increase health care professionals’ skills in delivering patient education.

On the other hand, health care professionals found some disadvantages of the patient education course. The disadvantages were mainly related to the scheduling problems of the course when most of the health care professionals worked in shifts and some of them were on vacation. In the future, it would be important to pay even more attention to planning the course to minimize these kinds of disadvantages.

Currently, continuing education is a vital part of the European Commission’s lifelong learning policy [10], and the costs of continuing vocational education are significant in European Union countries [11]. In addition, Finnish health and social services put a strong emphasis on continuing education. For example, in 2015, 69% of employees in social and health care (N=202,413) participated in continuing education with total costs of 48 million euros [93]. The costs of continuing education are based on, for example, travel costs and payment for substitutes for employees while they are studying. If at least some of the costs could be saved, by removing travel costs through Web-based education, for example, the savings could be millions per year for employers. In the future, Web-based courses will become increasingly important as younger health care professionals, who are accustomed to using computers and other information technology as a part of their daily activities, enter into the workforce.

### Limitations

The results of this study should be considered in the context of its limitations and strengths. First, the number of health care professionals was small, and measurements were done with a pre-post design without a control group, which limits the generalizability of the results. The original plan was to have a control group of other health care professionals from similar wards, but the number of health care professionals in the control group would have been very low; therefore, we decided to use data from the intervention group only. However, a quasi-experimental study design without a control group has been found to be a suitable method for medical informatics studies. Moreover, the design, with its pretest and multiple posttests, increases the validity of the method [52]. Second, dropout rates were high throughout the study, especially for the first follow-up measurement (17/33, 52%). This first follow-up measurement was conducted 8 weeks after baseline when the course was supposed to be completed. However, health care professionals had difficulties in recruiting patients to use MentalNet with them for the course. Therefore, after 8 weeks, the length of the course was delayed, which might have had an impact on measurements. However, the demographic characteristics of nonparticipating and participating health care professionals in the first follow-ups were similar. Therefore, although the dropouts in follow-ups can be assumed to be random, we can also assume that missing values were completely random and the assumptions for linear mixed models for repeated measures analyses were met.

Third, self-reported measurements were used to assess the impact of the course. More objective measurements could offer a more valid perspective on the impact of the course. For example, only the health care professionals were asked about the advantages and disadvantages of the Web-based course. The perspective of other stakeholders, such as patients [34], could widen the understanding of the real impact of the course in clinical practice [94]. In addition, we may ask whether our outcome measures were specific enough to measure the impact of the Web-based course for health care professionals. More studies are needed in this area.

Despite the study design used and the small sample size, we might still assume that health care professionals’ Web-based training has a positive impact on health care professionals’ self-efficacy. This finding is significant for health care leaders when evaluating the importance of education in the professional performance of health care workers [12,13], especially when considering Web-based education or other learning methods involving technological devices.

### Conclusions

Our study provides essential information on how a Web-based course can be useful to health care professionals as continuing education. The results of this study show that self-efficacy of health care professionals can be supported by Web-based continuing education. In addition, this kind of study, where the study task is for health care professionals to use a Web-based program with their patients, can improve the relationships between health care professionals and patients. The results of this study can be utilized when planning a Web-based course and trying to avoid difficulties with scheduling the course and recruiting the patients, as we experienced here. When offering a course in which health care professionals need to work with patients with schizophrenia, there should be enough time to recruit patients. In addition, shift work and vacations can cause challenges in maintaining the course. Therefore, an adequate amount of consideration should be spent on scheduling the course.

## Abbreviations

GSE: General Self-Efficacy Scale
SES: Self-Esteem Scale
TCI: Team Climate Inventory
